# Polarity-Dependent Asymmetric Distribution and MEX-5/6–Mediated Translational Activation of the *Era-1* mRNA in *C*. *elegans* Embryos

**DOI:** 10.1371/journal.pone.0120984

**Published:** 2015-03-30

**Authors:** Zoltán Spiró, Pierre Gönczy

**Affiliations:** Swiss Institute for Experimental Cancer Research (ISREC), School of Life Sciences, Swiss Federal Institute of Technology (EPFL) Lausanne, Lausanne, Switzerland; East Carolina University, UNITED STATES

## Abstract

The early *C*. *elegans* embryo is an attractive model system to investigate fundamental developmental processes. With the exception of *mex-3* mRNA, maternally contributed mRNAs are thought to be distributed uniformly in the one-cell embryo. Here, we report and characterize the striking distribution of the mRNA encoding the novel protein ERA-1. We found that *era-1* mRNA is enriched in the anterior of the one-cell embryo and present solely in anterior blastomeres thereafter. Although *era-1* is not an essential gene, we uncovered that *era-1* null mutant embryos are sensitive to slight impairment of embryonic polarity. We found that the asymmetric distribution of *era-1* mRNA depends on anterior-posterior polarity cues and on the *era-1* 3’UTR. Similarly to the *era-1* mRNA, the YFP-ERA-1 protein is enriched in anterior blastomeres. Interestingly, we found that the RNA-binding protein MEX-5 is required for *era-1* mRNA asymmetry. Furthermore, we show that MEX-5, together with its partially redundant partner MEX-6, are needed to activate *era-1* mRNA translation in anterior blastomeres. These findings lead us to propose that MEX-5/6–mediated regulation of *era-1* mRNA contributes to robust embryonic development.

## Introduction

Before zygotic transcription begins, restricted distribution of maternally provided mRNAs and their translational regulation are widely used mechanisms to orchestrate early developmental processes, including in the one-cell embryo (hereafter referred to as the zygote) (reviewed in [1–3]). Compared to most other metazoan organisms where this issue has been investigated, *C*. *elegans* stands out because apart from *mex-3* [4], no mRNA with a restricted distribution in the zygote has been reported to date.

The *C*. *elegans* zygote becomes polarized shortly after fertilization along the anterior-posterior (A-P) embryonic axis through the action of PAR proteins and associated components, which localize to the anterior (PAR-6, PAR-3, PKC-3) or posterior (PAR-2, PAR-1) cell cortex (reviewed in [5]). Further A-P asymmetries are generated as a result, including an anterior enrichment of the two partially redundant CCCH RNA-binding proteins MEX-5 and MEX-6, which function to reinforce polarity [6,7]. Such anterior enrichment requires phosphorylation of MEX-5 by the PAR-1 kinase, which results in increased MEX-5 diffusion in the posterior and thus to an A-P MEX-5 gradient [8]. As a result of proper A-P polarity, the zygote divides unequally, yielding the larger anterior cell AB and the smaller posterior cell P_1_. Thereafter, AB divides symmetrically, generating ABa and ABp, whereas P_1_ divides asymmetrically, resulting in EMS and P_2_.

Translational regulation plays an important role during *C*. *elegans* development past the two-cell stage. Thus, MEX-5/6, together with MEX-3 and SPN-4, regulate the translation of several mRNAs in anterior blastomeres, while POS-1 regulates that of mRNAs in posterior blastomeres [9–11]. In general, translational repressors and activators act by binding to the 3’UTR of target mRNAs. For example, translation of the uniformly localized mRNA encoding the Notch receptor GLP-1 is activated in ABa and ABp by binding of MEX-5, SPN-4 and MEX-3 to the *glp-1* 3’UTR, whereas it is repressed, also through the 3’UTR, by POS-1 in EMS and P_2_ [11,12]. Similarly, the homogenously distributed *zif-1*mRNA, which encodes an E3 ubiquitin protein ligase complex component, is also under such dual regulation, with MEX-5-mediated activation winning over POS-1-directed repression in anterior blastomeres, thereby giving rise to asymmetric ZIF-1 protein distribution [13].

Although the majority of maternally provided mRNAs exhibits uniform distribution in early *C*. *elegans* embryos (so-called class I mRNAs [14]), a few have been identified as being polarized in embryos past the 2-cell stage. Amongst them, class II mRNAs such as *cey-2*, *skn-1* or *pos-1* are distributed in a uniform manner in the zygote, but become enriched in the P-lineage thereafter following their preferential degradation in the other cells [10,14]. Single cell mRNA deep sequencing experiments revealed that 4 mRNAs are more abundant in P_1_ than in AB, whereas the converse is true for 13 other mRNAs [15]. Whether any of these mRNAs exhibits an asymmetric distribution in the zygote has not been addressed. Therefore, the *mex-3* mRNA, which is enriched on the anterior side in the zygote and present solely in anterior blastomeres thereafter, is the only mRNA known to exhibit a restricted distribution in the *C*. *elegans* zygote, and the mechanisms governing its distribution are not understood [4,9]

## Materials and Methods

### Worm strains and molecular biology

Wild type (N2) animals were maintained at 20–24°C, transgenic worms expressing YFP-ERA-1*[3’era-1]* UTR, YFP*[3’era-1]* and YFP-ERA-1*[3’pie-1]* at 24°C. All constructs were subcloned into pCFJ178 [15], which expresses the *C*. *briggsae unc-119* genomic sequence. The *yfp* gene with 3 introns [16] was fused to the *era-1* genomic sequence, from the start codon to the end of the 174 nt-long 3’UTR (*yfp-era-1[3’era-1*) or to the stop codon of the open reading frame (*yfp-era-1[3’pie-1]*). The 120 nt-long *pie-1* 3’UTR was used for *yfp-era-1[3’pie-1]*. Transgenic lines were generated by ballistic bombardment in *unc-119(ed3)* animals [17]; the designations of the resulting strains are as follows: GZ1146—*isIs101[pie-1*::*yfp-era-1[3'era-1] unc-119(+)*; GZ1280 -*isIs102[pie-1*::*yfp[3'era-1] unc-119(+)* and GZ1169—*isIs103[pie-1*::*yfp-era-1[3'pie-1] unc-119(+)*. Note that for the latter strain (GZ1169), phenotypically wild-type animals segregate ∼50% wild-type, ∼25% Uncs and ∼25% sterile animals with protruding vulva (Ste/Pvl). Because the Ste/Pvl phenotype was not suppressed by *gfp*(*RNAi*), we surmise that it does not stem from the presence of YFP-ERA-1 protein, but instead from disruption, through transgene insertion, of a gene important for vulva development. However, we cannot exclude the possibility that GZ1169 carries an extrachromosomal array. The *era-1(tm5852)* and *era-1(tm6426)* strains were provided by the National Bioresource Project (NBRP) and were maintained at 20 or 24°C. Bacterial feeding strains for *era-1*, *mex-5*, *par-3* were from the Vidal library [18], that for *par-2* from the Ahringer library [19]. The feeding clones against *mex-6* and *par-1* were generated by amplifying a region of *mex-6* (between position 241 and 1014) and *par-1* (between positions 1288 and 2395) cDNAs, which were then cloned into the L4440 vector [20]. Feeding RNAi was performed for 24 hours at 24°C, except for *par-1*(RNAi), where 48 hours incubation at 24°C was needed. Double depletion was achieved by mixing the IPTG-induced individual bacterial cultures in a 1:1 ratio. For RNAi by injection and by soaking, dsRNA targeting the entire *era-1* mRNA was synthesized with the SP6 and T7 *in vitro* synthesis kits (Promega). Soaking was performed with L3 animals for 24 hours at 20°C and the resulting embryos investigated 24 hours after recovery. A similar recovery period followed injection of L4 animals with *era-1* dsRNA. *era-1* RNAi by feeding was performed for 20–24 hours at 24°C. To partially deplete *par-2* or *par-3*, L4 animals were fed with bacteria expressing the relevant dsRNAs for 4 hours at 24°C and then transferred to plates containing OP50 at 20°C, after which the laid eggs were scored for embryonic viability.

### Live imaging

Dual time-lapse DIC and fluorescence imaging was performed on a Zeiss Axioplan 2 as described [21]. The motorized filter wheel, two external shutters, and the 1,392 x 1,040 pixels 12-bit Photometrics CoolSNAP ES2 were controlled by *μ*Manager [22]. Images were acquired with an exposure time of 10–100ms for the DIC and 300 ms for the fluorescence channel using the Zeiss Filter Set 10. Embryos were allowed to develop under the coverslip without imaging and snapshots were taken at the indicating times.

### Immunostaining

For immunostaining, embryos were fixed in methanol at −20°C for 30 minutes followed by incubating overnight at 4°C with primary antibodies. Primary mouse antibodies against α-tubulin (1/300; DM1A, Sigma) or GFP (1/100; MAB3580, Millipore) were used together with rabbit antibodies against GPA-16 (1/200, [23] or PAR-2 (1:200, [24]. Secondary antibodies were Alexa488-conjugated goat anti-mouse (1/500; Life Technologies, A11001) and Cy3-conjugated goat anti-rabbit (1/1000; Jackson ImmunoResearch, 711-165-152). Slides were counterstained with 1mg/ml Hoechst 33258 (Sigma) to visualize DNA. Images were acquired on an LSM700 confocal microscope (Zeiss) and processed in ImageJ.

For quantification of cell membrane enrichment, the fluorescence intensities of YFP and GPA-16 were measured along the membrane in the middle plane of the embryo and values expressed as a ratio between YFP and GPA-16 intensities.

### RT-qPCR

Embryos were bleached and total mRNA isolated using trizol-chloroform extraction [25]. 200 μg of total RNA was then reverse transcribed using poly-dT or random hexamer primers. Specific primer pairs were used to amplify the cDNA with the Power SYBR Green PCR mix (Applied Biosystems). The PCR reaction was performed in the 7900HT Fast Real-Time PCR System machine (Applied Biosystems) with the SDS2.4 software using 60°C for annealing and 72°C for elongation. The ΔΔC_T_ method was used to quantify mRNA levels relative to the reference gene, *act-1*. The primers were as follow:

*act-1* F – AGTCCGCCGGAATCCACGAG

*act-1* R – CTTGATCTTCATGGTTGATG

*yfp* F – GGGAACTACAAGACACGTGC

*yfp* R – GTGTCCAAGAATGTTTCC

### Digoxigenin *in situ* hybridization

Digoxigenin-labeled single stranded DNA probes were prepared using the PCR DIG Probe Synthesis Kit (Roche) following the manufacturer’s instructions. Briefly, the plasmid carrying the gene of interest served as template in a PCR-based reaction, with a reverse primer binding to the 3’UTR added together with the DIG-11-dUTP containing dNTP mix. Note that we did not linearize the plasmid when preparing the probes but used the circular form for the asymmetric PCR instead, reasoning that non-specific sequences in the probe mixture might help block non-specific binding sites. The resulting PCR product was ethanol-precipitated and resuspended in hybridization buffer (50% deionized formamide, 5x SSC, 100 μg/ml sonicated salmon testis DNA, 100 μg/ml yeast tRNA, 100 μg/ml heparin, 0.1% Tween-20) at a concentration of 50 ng/μl, boiled at 95°C for 75 minutes to reduce its size (Seydoux and Fire, 1995) and stored at −20°C.

The *in situ* hybridization protocol we developed is a combination of two previously available procedures [26,27]. Gravid hermaphrodites were bleached and the resulting embryos placed on a poly-D-lysine coated slide in a total volume of 7μl diluted in water. Then a coverslip was gently deposited on the embryos, and the freeze-crack method used to remove their eggshell. Subsequent steps were performed in a plastic slide holder containing 150 ml of the indicated solutions, unless otherwise stated. First, the slides were then submerged into −20°C methanol for 5 minutes and thereafter into sequential mixtures of methanol and Hepes-PBS-formaldehyde (HPF) solutions at 4°C (1x PBS, 75mM Hepes pH = 6.9, 0.03% EGTA, 1.5 mM MgSO_4_, 3.6% formaldehyde); ratios of water:methanol:Hepes-PBS-formaldehyde being first 50:15:35, then 50:25:25 and finally 50:35:15), for 2 minutes each at 4°C, before fixation in 3.6% formaldehyde in HPF solution for 20 minutes at 4°C [26]. Following a wash with PBS-Tween (0.1%, PBS-Tw) for 5 minutes at room temperature, Proteinase K (5.6 μg/ml) digestion was performed for 10 minutes at 24°C in PBS-Tw and then stopped by submerging the slides into 2 g/l glycine dissolved in PBS for 2 min followed by 2 washes in PBS-Tw for 2 minutes each. The slides were then re-fixed in HPF solution for 50 minutes at room temperature, washed twice with PBS-Tw for 5 minutes each, treated with 2 mg/ml glycine for 5 minutes and washed again once with PBS-T. Slides were then submerged into Formamide-SSC solution (50% formamide-5xSSC (pH = 7) -100 μg/ml heparin, 0.1% Tween-20), mixed with PBS-Tw in a 1:1 ratio for 10 minutes and thereafter in Formamide-SSC for another 10 minutes. Pre-hybridization was carried out in 50 μl hybridization buffer (50% deionized formamide 50%, 5xSSC, 100 μg/ml sonicated salmon testis DNA, 100 μg/ml yeast tRNA, 100 μg/ml, heparin 0.1% Tween 20) for 1 hour at 48°C, after which the probe was applied at a concentration of 40 ng/μl in 50 μl overnight, again at 48°C. To prevent evaporation, parafilm squares glued with rubber cement were mounted on the slides. The probe was washed off twice with Formamide-SSC:PBS-Tw (1:1) for 10 minutes and then 4 times using 0.1% Chaps in 0.8x PBS at 48°C for 20 minutes each [26], after which a blocking step was included using 0.1%BSA—0.1%Triton-X in 1xPBS (PBS-BSA-Tx) for 1.5 hours at room temperature. The alkaline-phosphatase (AP)-conjugated anti-DIG antibody (Roche) was diluted 1:2000 in 1x PBS and incubated at 4°C overnight. The slides were washed 5 times with PBS-BSA-Tx and twice with staining buffer (100 mM Tris-HCl (pH = 9.5), 100mM NaCl, 5mM MgCl_2_, 1mM Levamisole, 0.1% Tween-20); the signal was developed using a mixture of 333 μg/ml NBT (Roche) and 70 μg/ml BCIP (AppliChem) for 20–60 minutes at room temperature. The slides were then washed once with PBS-Tw for 5 minutes at room temperature, stained with 1mg/ml Hoechst 33258 (Sigma) in PBS-Tw, then washed once with PBS, mounted and stored at 4°C. All *in situ* hybridization experiments were performed at least twice and representative embryos are shown in the figure panels. Control embryos were included on one side of the slide in each instance.

Quantification of mRNA levels was performed by measuring mean intensities in the cytoplasm of AB and P_1_ (A, P), of the background outside the embryos (B), as well as in later stage embryos, where the hybridization did not give any signal (C). The value B was subtracted from A, P and C, and the resulting corrected values used to determine the ratios A/C and P/C, which are reported in the figures with arbitrary units (A.U.). In some instances, one-cell embryo shown in the figures were surrounded by multicellular embryos possessing high Hoechst signal intensities, leading to an overflow of the blue signal onto the embryo of interest. In these cases, the background was subtracted and the Hoechst image divided by its Gaussian blurred value, resulting in clearing off the non-pertinent signal. Then the brightness was enhanced for clearer visualization of the relevant DNA signal.

## Results and Discussion

### The *era-1* mRNA is enriched in the anterior of the *C*. *elegans* zygote and in anterior blastomeres thereafter

To determine if there are localized mRNAs in the zygote apart from that of *mex-3*, we analyzed the distribution of the 11’237 mRNAs available in an *in situ* hybridization database (Nematode Expression Database (NEXTDB), http://nematode.lab.nig.ac.jp).This database contains typically 4–6 images, each with 7–8 embryos at different stages of development. We first analyzed these images at low magnification; when a non-uniform distribution seemed to be spotted in early stage embryos in this manner, the images were magnified to further assess mRNA distribution. This analysis led us to uncover that the transcript of the uncharacterized open reading frame W02F12.3 displays a suggestive distribution, with a bias towards the anterior of the zygote and of embryos in subsequent stages of embryogenesis. We dubbed this gene *era-1* (for embryonic mRNA anterior) and investigated it further.

**Fig 1:**
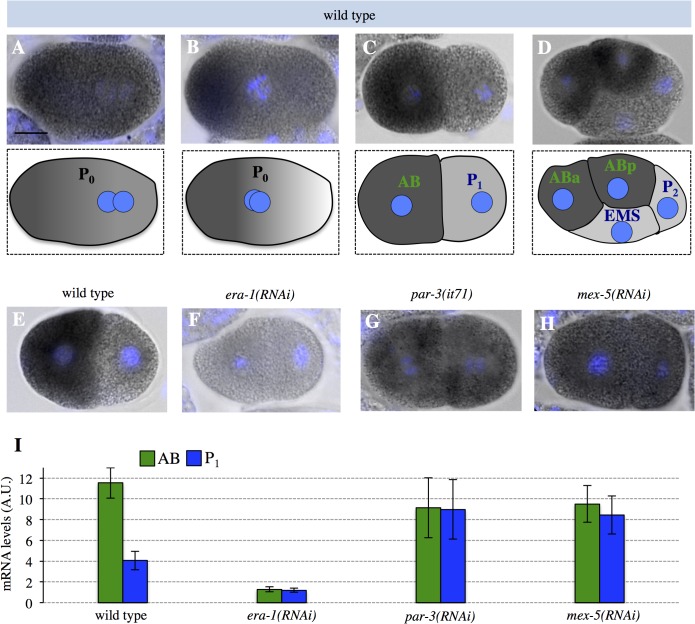
*era-1* mRNA is enriched on the anterior of early *C*. *elegans* embryos. **A-D** *era-1* mRNA distribution in the wild-type zygote (P_0_), in prophase (A) or prometaphase (B), as well as in 2-cell (C) and 4-cell (D) embryos. Here and in other figures, the mRNA appears dark grey, the DNA light blue and scale bars represent 10 microns. **E-H** *era-1* mRNA distribution in control (E), *era-1(RNAi)* (F), *par-3(RNAi)* (G) and *mex-5(RNAi)* (H) 2-cell embryos. **I** Corresponding quantifications of mRNA levels in AB and in P_1_ (Materials and Methods). Number of embryos quantified: wild type, n = 9; *era-1(RNAi)*, n = 12; *par-3(RNAi)*, n = 10; *mex-5(RNAi)*, n = 7. In this and other figures, experiments were performed at least twice, mean values ± standard deviations (SD) are shown, with exact values given in S1 Table. Statistical analysis was performed using unpaired Student’s t-test to compare mRNA levels in AB versus P_1_, yielding the following p-values: wild type—p = 5.67×10^−10^, *era-1(RNAi)*, p = 0.326; *par-3(RNAi)*, p = 0.907; *mex-5(RNAi)*, p = 0.287.

We ascertained the asymmetric distribution of *era-1* mRNA by digoxygenin-alkaline phosphatase *in situ* hybridization. We found that asymmetric enrichment of *era-1* mRNA becomes apparent during prophase of the first cell cycle (Fig. 1A-B). Moreover, *era-1* mRNA is enriched in the anterior blastomere AB compared to the posterior blastomere P_1_ in two-cell embryos (Fig. 1C, 1I). This difference persists when comparing the AB daughters ABa and ABp with the P_1_ daughters EMS and P_2_ (Fig. 1D). No signal is detected in *era-1(RNAi)* embryos, indicating probe specificity (Fig. 1F, 1I).

Intriguingly, *era-1* is not among the genes identified by single cell mRNA sequencing as being enriched in AB versus P_1_ [28]. Conversely, of the 13 mRNAs identified in that study as being enriched in AB versus P_1_, only *mex-3* mRNA shows a clear asymmetry in the Nematode Expression Database. Another 6 mRNAs appear to be uniformly distributed, 5 have not been tested and 1 was not analyzable due to an oversaturated signal. Therefore, mRNA sequencing and large-scale *in situ* hybridization methods provide complementary means to identify mRNAs with an asymmetric distribution in *C*. *elegans* embryos.

### Structure and function of *era-1*

The *era-1* gene is predicted to encode a 388 amino acids long polypeptide, with no clear protein domains (S1A Fig.). We found distant relatives of ERA-1 in other nematodes (S1B Fig.), although they are not *bona fide* orthologues, since performing BLAST analysis with them against the *C*. *elegans* proteome did not identify ERA-1 as the first hit, indicating fast divergence from a common ancestor.

We set out to address the function of *era-1* in *C*. *elegans*. A comprehensive RNAi-based functional genomic screen did not reveal a requirement for *era-1* [29]. Accordingly, we found that performing *era-1*(*RNAi*) by injection, soaking or feeding did not result in lethality or in an obvious phenotype in the resulting progeny. Furthermore, we found that the deletion alleles *era-1(tm5852)* and *era-1(tm6426)* are viable and fertile and do not exhibit an apparent phenotype in the embryo (S1A, S1C Fig.). We conclude that *era-1* is not an essential gene under standard laboratory conditions.

Because of the intriguing polarized distribution of the *era-1* mRNA, as well as of the rapid sequence divergence amongst nematodes, we explored whether *era-1* may exhibit synthetic lethality with genes required for embryonic polarity in *C*. *elegans*. Intriguingly, we found that *era-1(tm6426)* embryos are indeed more sensitive than the wild-type to partial RNAi-mediated depletion of PAR-3 or PAR-2 (S1C Fig.). Whereas future work will be needed to determine the mechanism underlying such synthetic lethality, we conclude that ERA-1 plays a non-essential role in ensuring robust embryonic development.

### *era-1* mRNA distribution requires MEX-5 and the *era-1* 3’UTR

We sought to address what regulates the remarkable distribution of the *era-1* mRNA. First, we asked if anterior enrichment is polarity-dependent. As shown in Fig. 1G and 1I, we found that this is the case, since *era-1* mRNA distribution is uniform in *par-3(RNAi)* embryos. What other *trans*-acting factor could govern such anterior enrichment? The anteriorly localized CCCH finger protein MEX-5 seemed a plausible candidate since it is likewise enriched in the anterior of one-cell stage embryos [9]. Accordingly, we found that *era-1* mRNA is localized in a uniform manner in *mex-5(RNAi)* embryos (Fig. 1H, 1I). Although this could merely reflect a role for MEX-5 in polarity establishment, evidence shown below suggests that MEX-5 plays a more direct role in regulating *era-1* mRNA.

**Fig 2:**
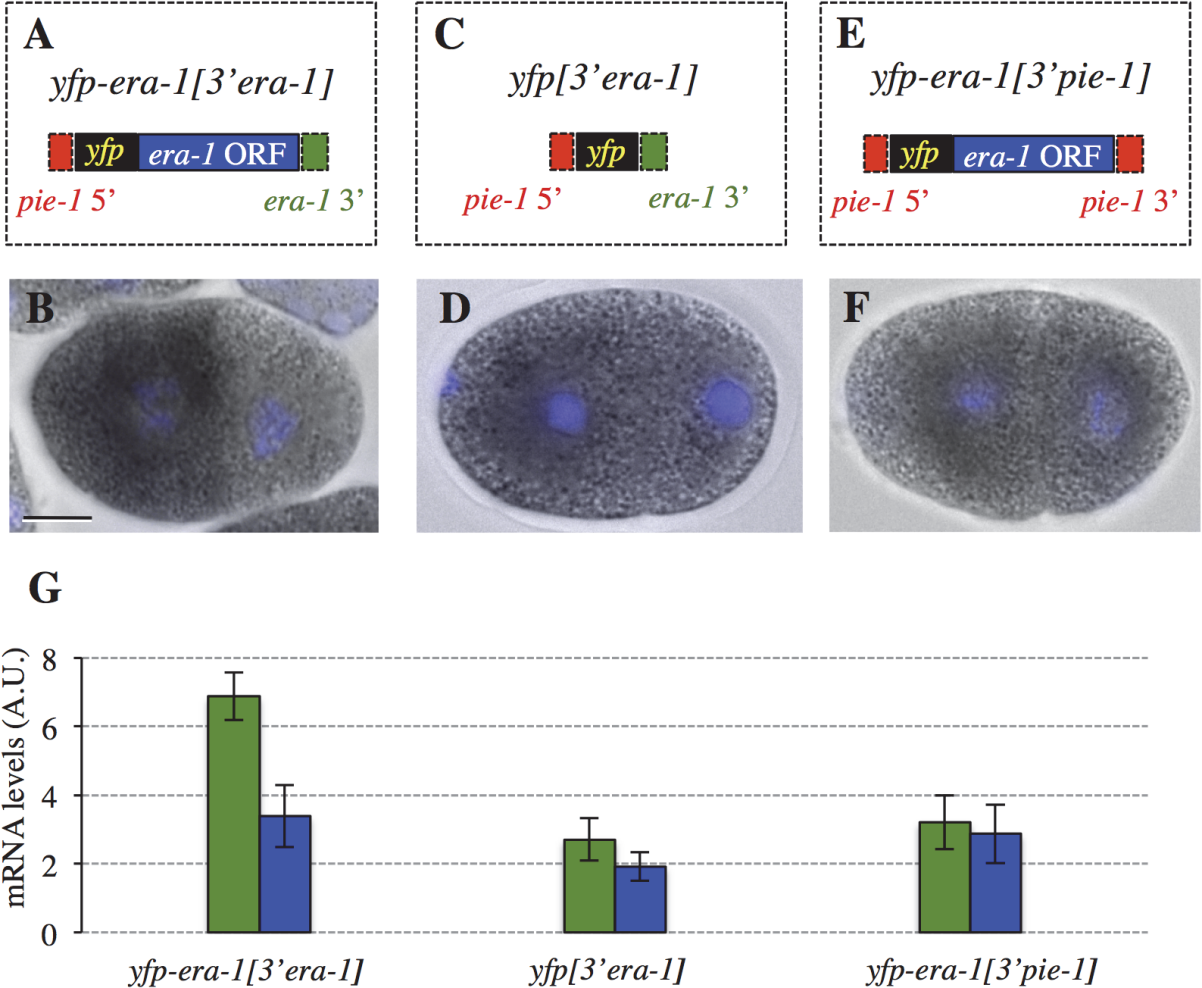
*yfp*-tagged *era-1* mRNA recapitulates endogenous *era-1* mRNA distribution in a 3’UTR-dependent manner. **A, C, E** Schematic representation of *yfp-era-1[3’ era-1]* (A), *yfp[3’era-1]* (C) and *yfp-era-1[3’pie-1]* (E) constructs. **B, D, F** Localization of these constructs in 2-cell embryos. **G** Corresponding quantifications of mRNA levels in AB and in P_1_ (Materials and Methods). Number of embryos quantified: *yfp-era-1[3’era-1]*, n = 9;. *yfp[3’era-1]*, n = 8; *yfp-era-1[3’pie-1]*, n = 10. Statistical analysis was performed using unpaired Student’s t-test to compare mRNA levels in AB versus P_1_, yielding the following p-values: *yfp-era-1[3’ era-1]*, p = 1.03×10^−7^; *yfp[3’era-1]*, p = 2×10^−4^; *yfp-era-1[3’pie-1]*, p = 0.362. Note that anterior enrichment of *yfp-era-1[3’era-1]* is somewhat less pronounced than that of endogenous *era-1* mRNA, perhaps because the *era-1* 5’UTR contributes to mRNA distribution or because the presence of *yfp* perturbs localization.

To identify *cis*-acting element regulating *era-1* mRNA distribution, we fused the sequence coding for the yellow fluorescent protein (YFP) to the *era-1* genomic sequence (exons, introns and 3’UTR) in an expression vector driven by the *pie-1* promoter (yielding *yfp-era-1[3’era-1]*, Fig. 2A). We generated a corresponding transgenic line and performed *in situ* hybridization against *yfp* mRNA. We found that the *yfp-era-1[3’era-1]* mRNA is also enriched in the anterior of the zygote and inherited by anterior blastomeres thereafter (Fig. 2B, 2G).

Sequences in the 3’UTR are responsible for localizing many mRNAs [30,31]. To test the contribution of the *era-1* 3’UTR in *era-1* mRNA distribution, we generated transgenic animals expressing *yfp* fused directly to the *era-1* 3’UTR (yielding *yfp[3’era-1]*, Fig. 2C, 2D). As shown in Fig. 2G, our analysis uncovered a significant anterior bias of *yfp[3’era-1]* mRNA, although the extent of enrichment in AB versus P_1_ is less pronounced than with *yfp-era-1[3’era-1]* (compare Fig. 2D with 2B). We conclude that the *era-1* 3’UTR, while not being sufficient for, contributes to mRNA anterior distribution, with the help of elements located in the coding sequence.

To test if the *era-1* 3’UTR is necessary for anterior mRNA distribution, we modified *yfp-era-1[3’era-1]* by replacing the *era-1* 3’UTR with the *pie-1* 3’UTR (yielding *yfp-era-1[3’pie-1]*, Fig. 2E), and generated a corresponding transgenic line. Strikingly, we found that the *yfp-era-1[3’pie-1]* mRNA localizes in a homogenous manner, with no difference between AB and P_1_ (Fig. 2F, 2G). Therefore, the *era-1* 3’UTR is necessary for anterior mRNA distribution.

### YFP-ERA-1 protein is enriched in anterior blastomeres

**Fig 3:**
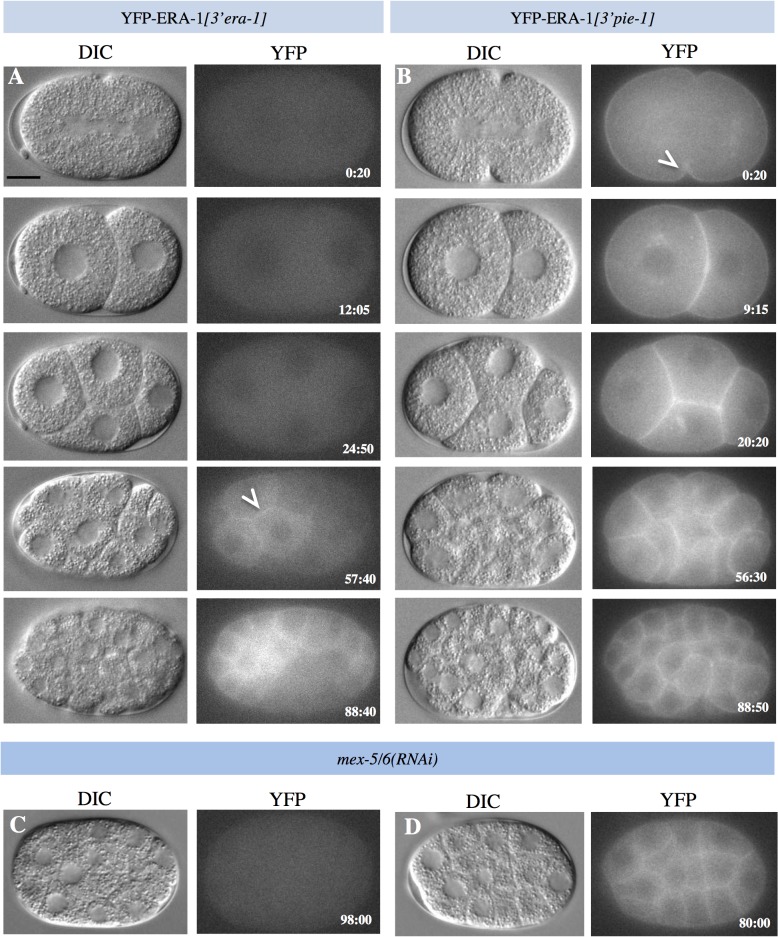
YFP-ERA-1 is enriched in anterior blastomeres in a 3’UTR- and MEX-5/6-dependent manner. **A-B** Snapshots from dual DIC and fluorescent time-lapse microscopy recordings of embryos expressing YFP-ERA-1*[3’era-1]* (A) and YFP-ERA-1*[3’pie-1]* (B). Time indicated in min:sec, with t = 0 corresponding to cytokinesis onset in P_0_. Arrowheads points to YFP signal at the cell cortex. Brightness and contrast were lowered in panel B to avoid saturating the signal. **C-D** Snapshots from late stage *mex-5/6(RNAi)* embryos expressing YFP-ERA-1*[3’era-1]* (C) and YFP-ERA-1*[3’pie-1]* (D).

**Fig 4:**
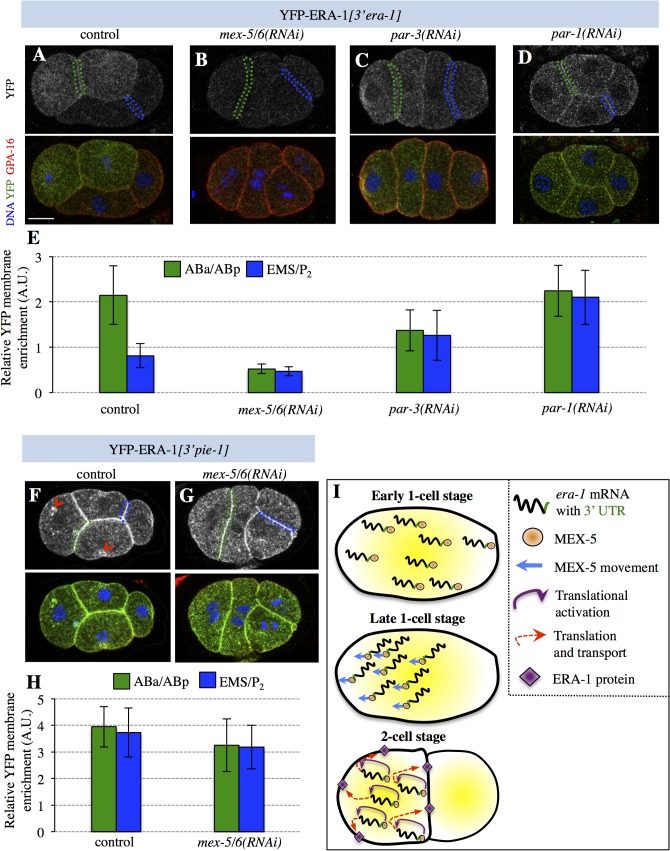
MEX-5/6-mediated translational activation of *era-1*. **A-D** 4-cell embryos expressing YFP-ERA-1*[3’ era-1]* in control (A), *mex-5/6(RNAi)* (B), *par-3(RNAi)* (C) and *par-1(RNAi)* (D) conditions, stained with antibodies against GFP (green) and GPA-16 (red); DNA is viewed in blue. **E** Signal intensities at the cell-cell boundaries of ABa/ABp and EMS/P_2_ relative to GPA-16 (Materials and Methods). Number of embryos quantified: control, n = 10; *mex-5/6(RNAi)*, n = 10; *par-3(RNAi)*, n = 9; *par-1*(*RNAi*), n = 10. Statistical analysis was performed using unpaired Student’s t-test to compare control with RNAi conditions for both anterior and posterior blastomeres, yielding the following p-values: *mex-5/6(RNAi)*, anterior: p = 4.42×10^–7^; posterior: p = 1.46×10^–3^; *par-3(RNAi)*, anterior: p = 9.32×10^–3^; posterior: p = 0.04; *par-1(RNAi)*, anterior: p = 0.741; posterior: p = 1.81×10^–5^. **F-G** 4-cell embryos expressing YFP-ERA-1*[3’ pie-1]* in control (F) or upon *mex-5/6(RNAi)* (G). Red arrowheads in F indicate cytoplasmic foci resembling endomembranes, suggesting that ERA-1 traffics through endosomal compartments. **H** Signal intensities at the cell-cell boundaries of ABa/ABp and EMS/P_2_ relative to GPA-16 (Materials and Methods). Number of embryos quantified: control, n = 10*; mex-5/6(RNAi)*, n = 8. Statistical analysis was performed using unpaired Student’s t-test to compare control with *mex-5/6*(*RNAi*) for both anterior and posterior blastomeres, yielding the following p-values: *mex-5/6 (RNAi)*, anterior: p = 0.113; posterior: p = 0.205. **I** Working model of MEX-5/6-mediated control of *era-1* mRNA asymmetric distribution in P_0_ and translational activation in 2-cell embryos; see also main text.

To address whether the anterior enrichment of *era-1* mRNA is accompanied by a likewise protein distribution, we analyzed embryos expressing *yfp-era-1[3’era-1]* using dual differential interference contrast (DIC) and fluorescent time-lapse microscopy. This revealed that YFP-ERA-1*[3’era-1]* protein can be detected starting from the ∼10 cell stage solely in descendants of the anterior blastomere AB (Fig. 3A). We also performed immunofluorescence analysis using antibodies against YFP that offer a more sensitive detection means. We thus found that YFP-ERA-1*[3’era-1]* is distributed uniformly in the zygote and becomes enriched in the AB cell (S1D, S1E Fig.), an anterior bias that becomes more pronounced in 4-cell stage embryos (Fig. 4A, 4E). Intriguingly, we noted also that YFP-ERA-1*[3’era-1]* is slightly enriched on the cell cortex, something that is most apparent at the boundary between anterior blastomeres (Fig. 4A), raising the possibility that ERA-1 is a cortical protein. To address if the anterior enrichment of YFP-ERA-1*[3’era-1]* protein is dependent on A-P polarity, we analyzed embryos depleted of PAR-3. As anticipated from the uniform distribution of the *era-1* mRNA in such embryos (see Fig. 1I), we found that YFP-ERA-1*[3’era-1]* is present at comparable levels in all blastomeres of *par-3*(*RNAi*) embryos (Fig. 4C, 4E).

To test if the *era-1* 3’UTR is important for restricting the fusion protein to anterior blastomeres, we examined embryos expressing *yfp-era-1[3’pie-1]*. Interestingly, we found that YFP-ERA-1*[3’pie-1]* protein is present in a uniform manner in the zygote and thereafter (Figs. 3B, 4F, 4H). We conclude that the *era-1* 3’UTR is important for restricting the presence of the fusion protein to anterior blastomeres during embryogenesis. Moreover, we noted that YFP-ERA-1*[3’pie-1]* protein levels are much higher than those of YFP-ERA-1*[3’era-1]*, even in anterior blastomeres (compare Figs. 3A with 3B and 4A with 4F). This is despite *yfp-era-1[3’pie-1]* mRNA levels being lower than those of *yfp-era[3’era-1]* (Fig. 2G). Because more protein is present already in the newly fertilized zygote when comparing YFP-ERA-1*[3’pie-1]* with YFP-ERA-1*[3’era-1]* (compare Fig. 3B with 3A, top row), our findings indicate also that the *era-1* 3’UTR prevents translation during oogenesis.

### MEX-5/6 positively regulates *era-1* translation via the *era-1* 3’UTR

Because both *era-1* mRNA and YFP-ERA-1 protein are enriched in anterior blastomeres, the distribution of the protein could be dictated merely by that of the mRNA. Alternatively or in addition, translational activation could also occur in anterior blastomeres. What factors could be responsible for such putative activation? MEX-5 and MEX-6 activate translation of mRNAs such as *mex-3* and *glp-1* in anterior blastomeres [9]. Compatible with this possibility, we found three MEX-5/6 target consensus sequences (i.e. ≥6 U out of 13 nucleotides [12] in the *era-1* 3’UTR, as well as four additional ones in the *era-1* coding sequence (S2A Fig.).

We depleted MEX-5/6 to explore whether they may regulate *era-1* mRNA translation. Strikingly, we found that YFP-ERA-1*[3’era-1]* protein levels are greatly reduced in *mex-5/6(RNAi)* embryos (Figs. 3C, 4B, 4E). RT-qPCR analysis revealed a slight but statistically non-significant decrease of mRNA levels (S2B Fig.), suggesting that the reduced YFP signal is not due to mRNA degradation. Given that MEX-5/6 also play a role downstream of the PAR proteins in establishing A-P polarity [9], and given that *era-1* mRNA distribution is homogenous in *mex-5(RNAi)* embryos, in principle such uniform distribution could merely reflect polarity defects. However, we view this as unlikely, because YFP-ERA-1*[3’era-1]* levels are lower in *mex-5/6(RNAi)* embryos than in *par-3*(*RNAi*) embryos, in which polarity is likewise affected (Fig. 4B, 4C, 4E). Of note, depletion of the translational regulator MEX-3 did not affect YFP-ERA-1*[3’era-1]* protein levels (S2C Fig.), indicating specificity of the effect incurred following MEX-5/6 depletion. Furthermore, we found that the levels of YFP-ERA-1*[3’pie-1]* protein are unaffected in *mex-5/6*(*RNAi)* embryos (Figs. 3D, 4G), indicating that translational activation occurs through the *era-1* 3’UTR.

We reasoned that if MEX-5/6 promote translational activation of *era-1* mRNA as suggested by the above experiments, posterior YFP-ERA-1*[3’era-1]* protein levels should be higher in embryos depleted of PAR-1, in which MEX-5/6 activity is increased in the posterior [8]. As shown in Fig. 4D and 4E, we found this to be the case indeed. Overall, these observations lead us to conclude that MEX-5/6 are needed to activate the translation of *era-1* mRNA through its 3’UTR during embryogenesis.

## Conclusions

Our findings suggest the following working model for the regulation of *era-1* mRNA and protein asymmetric distribution (Fig. 4I). In this model, *era-1* mRNA anterior enrichment is regulated by A-P polarity cues, MEX-5/6 and the *era-1* 3’UTR. MEX-5/6 could potentially transport *era-1* mRNA to the anterior, giving rise to its asymmetric distribution (Fig. 4I). Furthermore, MEX-5/6 act through the 3’UTR, directly or indirectly, to activate translation of the *era-1* mRNA in the embryo. These findings provide novel insights into the mechanisms by which mRNA distribution coupled with translational regulation contribute to robust development of *C*. *elegans* embryos.

## Supporting Information

S1 Fig The *C*. *elegans* specific *era-1* gene contributes to robust embryonic development. **A.** Schematic of the wild-type *era-1* gene and of *era-1(tm5852)* and *era-1(tm6426)* mutant alleles, with an indication of premature STOP codons (lack of translation is depicted in grey). Analysis with Scanprosit [1] failed to identify clear protein domains in ERA-1. **B.** Phylogenetic relationships of ERA-1-related proteins amongst select nematodes identified by Blast search (Ensembl). The tree was built with the Clustal W2 software (http://www.ebi.ac.uk/Tools/phylogeny/clustalw2_phylogeny/) on sequence alignment performed with the MAFFT server (http://mafft.cbrc.jp/alignment/server/). The values indicate the length of the branch leading to the previous node and show the number of substitutions as a proportion of the alignment length. **C.** Embryonic lethality following partial depletion of PAR-3 or PAR-2 in wild type and *era-1(tm6426)* worms. >800 embryos were scored in 8 independent experiments for *par-3(RNAi)*, >200 embryos in 3 independent experiments for *par-2(RNAi)*. Note that no enhancement was observed upon *glp-1(RNAi)* and *apx-1(RNAi)* as compared to wild type, indicating that the enhanced lethality observed with *par-3*(*RNAi*) and *par-2*(*RNAi*) is not a generic effect in response to RNAi. Statistical analysis was performed using unpaired Student’s t-test to compare embryonic lethality in wild type versus *era-1(tm6426)* in the indicated conditions, yielding the following p-values: partial *par-3(RNAi)*, p = 4.6×10^−3^; partial *par-2(RNAi)*, p = 0.048. **D-E.** YFP-ERA-1*[3’era-1]* protein at telophase of the first cell division (D) and in the 2-cell stage (E). The upper images show the YFP signal alone, the lower ones the merge. Scale bar represents 10 microns.
1. de Castro E, Sigrist CJ, Gattiker A, Bulliard V, Langendijk-Genevaux PS, Gasteiger E, et al. (2006) ScanProsite: detection of PROSITE signature matches and ProRule-associated functional and structural residues in proteins. Nucleic acids research 34: W362–365.
(PDF)

S2 Fig MEX-5-dependent translational regulation of *era-1* mRNA via potential MEX-5 binding sites. **A.** Putative MEX-5 binding sites (red) along the *era-1* exons (blue) and the 3’UTR (green). The sequences of the regions numbered 1–7 is shown below in red. **B.** mRNA levels of *yfp-era-1[3’era-1]*, *yfp[3’era-1]* and *yfp-era-1[3’pie-1]* measured by RT-qPCR with or without MEX-5 depletion (Materials and Methods). Values are shown relative to *act-1* mRNA. The experiment was performed 3 times. Average values are indicated with error bars representing the standard error of the mean. Statistical analysis was performed using unpaired Student’s t-test to compare mRNA levels in control and *mex-5*(*RNAi*) conditions, yielding the following p-values: *yfp-era-1[3’era-1]*, p = 0.429; *yfp[3’era-1]*, p = 0.234, and *yfp-era-1[3’pie-1]*, p = 0.284. **C.** Quantification of membrane enrichment relative to GPA-16 in 4-cell stage embryos expressing YFP-ERA-1*[3’era-1]* upon *mex-5* or *mex-3* depletion, as well as control embryos (Materials and Methods). Number of embryos quantified: control, n = 11; *mex-5(RNAi)*, n = 7; *mex-3(RNAi)*, n = 7. Statistical analysis was performed using unpaired Student’s t-test to compare control with RNAi conditions for both anterior and posterior blastomeres, yielding the following p-values: *mex-5(RNAi)*, anterior: p = 0.021; posterior: p = 0.012; *mex-3(RNAi)*, anterior: p = 0.465; posterior: p = 0.124 Note that although relative levels (i.e. levels of YFP-ERA-1*[3’era-1]* versus those of GPA-16) are lower than in Fig. 4E due to variation of signal intensities from experiment to experiment, the ratio of the anterior and posterior YFP-ERA-1*[3’era-1]* signals is comparable between the two experiments.
(PDF)

S1 Table Exact values and statistical analysis. The designation of the experiment (first column), the nature of the measurement (second column), the genotype/RNAi condition (A: anterior side, P: posterior side) (third column), the actual value (fourth column), the number of embryos or experiments analyzed (fifth column), the p-value using unpaired Student’s t-test (sixth column) and the corresponding Figure (last column) are reported.
(PDF)
