# Supplementary figures and images for: Polarity-Dependent Asymmetric Distribution and MEX-5/6–Mediated Translational Activation of the Era-1 mRNA in C. elegans Embryos
Source: PLoS One. 2015 Mar 30;10(3):e0120984. doi: 10.1371/journal.pone.0120984 (PMC4378847; doi:10.1371/journal.pone.0120984)

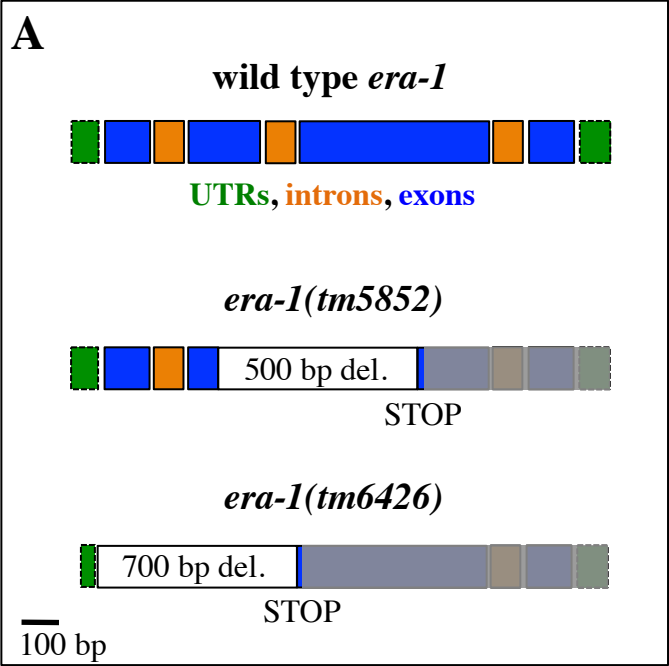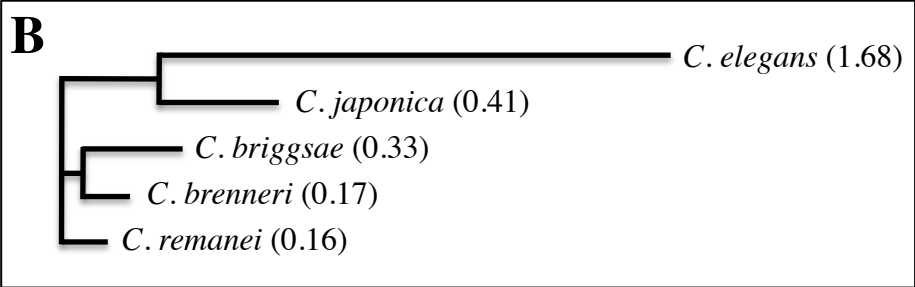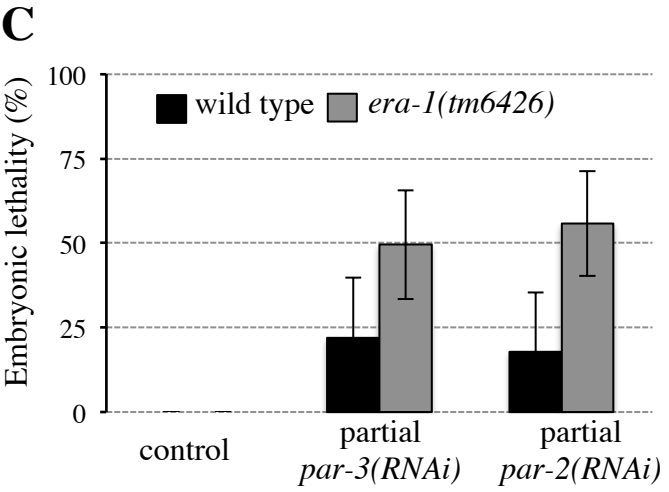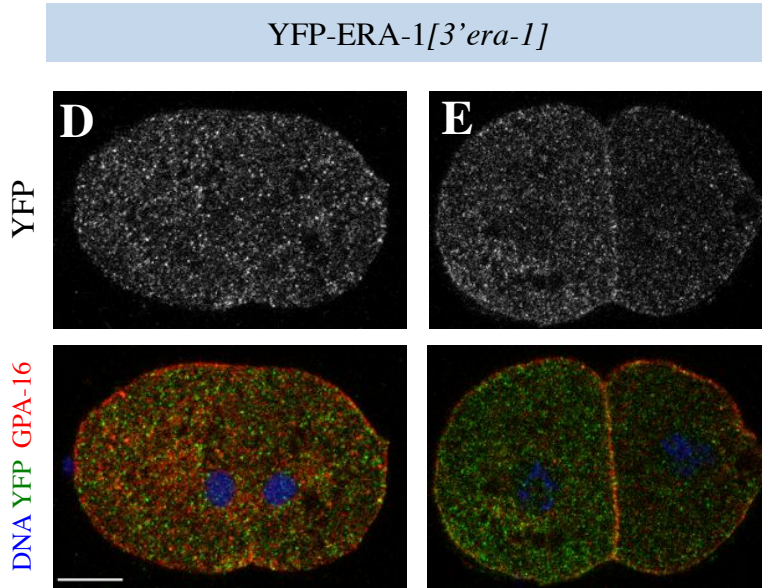

Supplement: S1 Fig — A. Schematic of the wild-type era-1 gene and of era-1(tm5852) and era-1(tm6426) mutant alleles, with an indication of premature STOP codons (lack of translation is depicted in grey). Analysis with Scanprosit [1] failed to identify clear protein domains in ERA-1. B. Phylogenetic relationships of ERA-1-related proteins amongst select nematodes identified by Blast search (Ensembl). The tree was built with the Clustal W2 software (http://www.ebi.ac.uk/Tools/phylogeny/clustalw2_phylogeny/) on sequence alignment performed with the MAFFT server (http://mafft.cbrc.jp/alignment/server/). The values indicate the length of the branch leading to the previous node and show the number of substitutions as a proportion of the alignment length. C. Embryonic lethality following partial depletion of PAR-3 or PAR-2 in wild type and era-1(tm6426) worms. >800 embryos were scored in 8 independent experiments for par-3(RNAi), >200 embryos in 3 independent experiments for par-2(RNAi). Note that no enhancement was observed upon glp-1(RNAi) and apx-1(RNAi) as compared to wild type, indicating that the enhanced lethality observed with par-3(RNAi) and par-2(RNAi) is not a generic effect in response to RNAi. Statistical analysis was performed using unpaired Student’s t-test to compare embryonic lethality in wild type versus era-1(tm6426) in the indicated conditions, yielding the following p-values: partial par-3(RNAi), p = 4.6×10^−3^; partial par-2(RNAi), p = 0.048. D-E. YFP-ERA-1[3’era-1] protein at telophase of the first cell division (D) and in the 2-cell stage (E). The upper images show the YFP signal alone, the lower ones the merge. Scale bar represents 10 microns. 1. de Castro E, Sigrist CJ, Gattiker A, Bulliard V, Langendijk-Genevaux PS, Gasteiger E, et al. (2006) ScanProsite: detection of PROSITE signature matches and ProRule-associated functional and structural residues in proteins. Nucleic acids research 34: W362–365. (PDF) [file pone.0120984.s001.pdf]

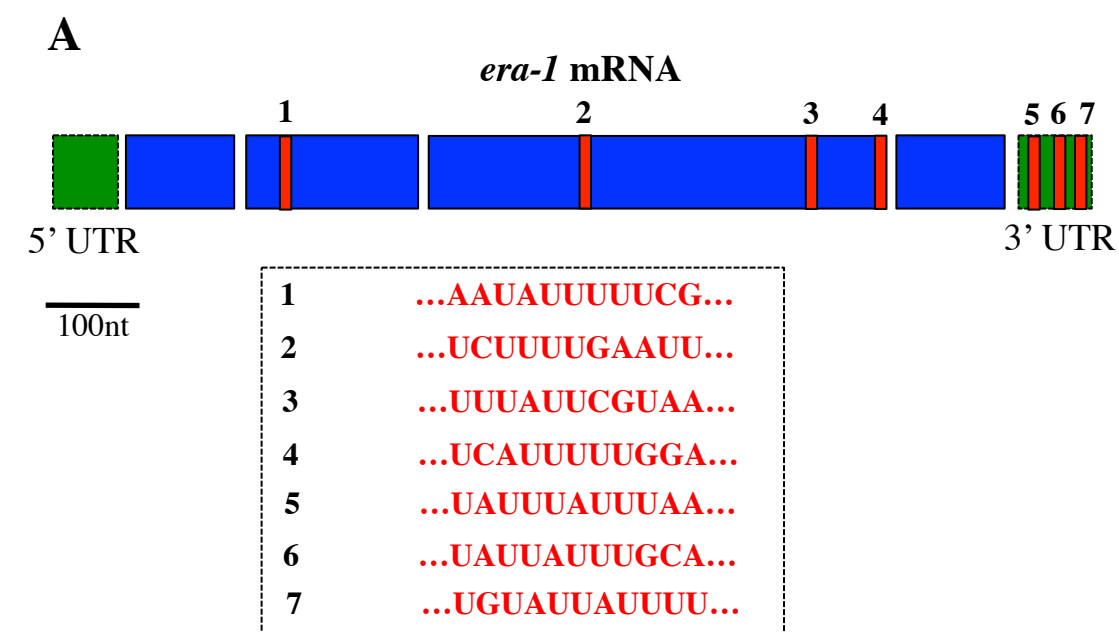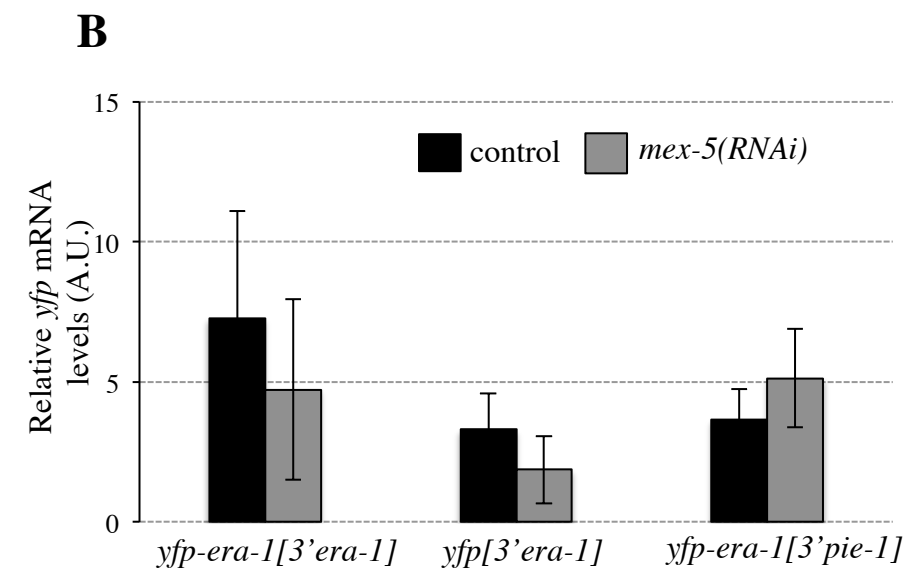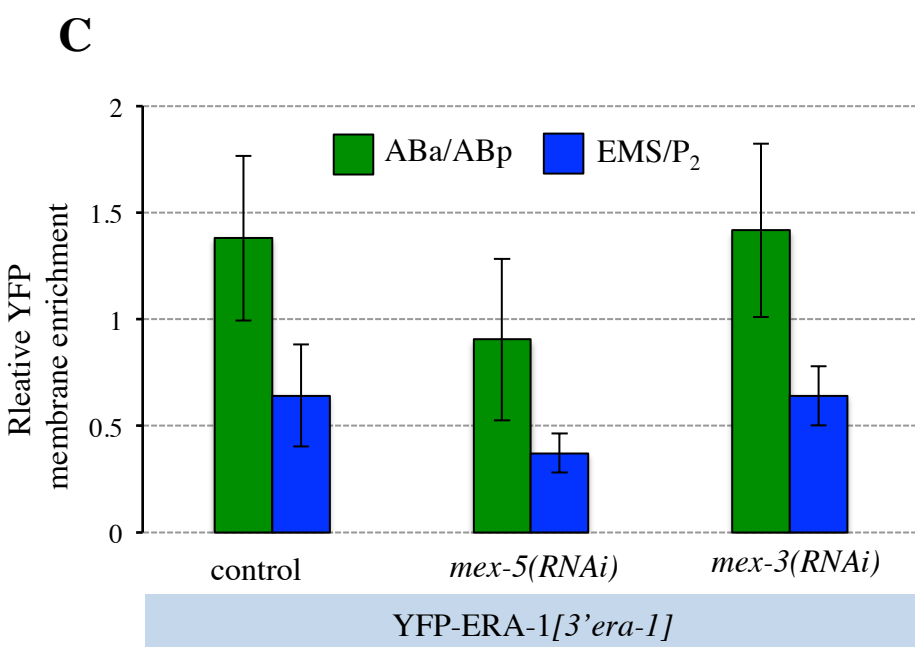

Supplement: S2 Fig — A. Putative MEX-5 binding sites (red) along the era-1 exons (blue) and the 3’UTR (green). The sequences of the regions numbered 1–7 is shown below in red. B. mRNA levels of yfp-era-1[3’era-1], yfp[3’era-1] and yfp-era-1[3’pie-1] measured by RT-qPCR with or without MEX-5 depletion (Materials and Methods). Values are shown relative to act-1 mRNA. The experiment was performed 3 times. Average values are indicated with error bars representing the standard error of the mean. Statistical analysis was performed using unpaired Student’s t-test to compare mRNA levels in control and mex-5(RNAi) conditions, yielding the following p-values: yfp-era-1[3’era-1], p = 0.429; yfp[3’era-1], p = 0.234, and yfp-era-1[3’pie-1], p = 0.284. C. Quantification of membrane enrichment relative to GPA-16 in 4-cell stage embryos expressing YFP-ERA-1[3’era-1] upon mex-5 or mex-3 depletion, as well as control embryos (Materials and Methods). Number of embryos quantified: control, n = 11; mex-5(RNAi), n = 7; mex-3(RNAi), n = 7. Statistical analysis was performed using unpaired Student’s t-test to compare control with RNAi conditions for both anterior and posterior blastomeres, yielding the following p-values: mex-5(RNAi), anterior: p = 0.021; posterior: p = 0.012; mex-3(RNAi), anterior: p = 0.465; posterior: p = 0.124 Note that although relative levels (i.e. levels of YFP-ERA-1[3’era-1] versus those of GPA-16) are lower than in Fig. 4E due to variation of signal intensities from experiment to experiment, the ratio of the anterior and posterior YFP-ERA-1[3’era-1] signals is comparable between the two experiments. (PDF) [file pone.0120984.s002.pdf]
