# Supplementary material for: Polarity-Dependent Asymmetric Distribution and MEX-5/6–Mediated Translational Activation of the Era-1 mRNA in C. elegans Embryos
Source: PLoS One. 2015 Mar 30;10(3):e0120984. doi: 10.1371/journal.pone.0120984 (PMC4378847; doi:10.1371/journal.pone.0120984)
Supplement: S1 Table — The designation of the experiment (first column), the nature of the measurement (second column), the genotype/RNAi condition (A: anterior side, P: posterior side) (third column), the actual value (fourth column), the number of embryos or experiments analyzed (fifth column), the p-value using unpaired Student’s t-test (sixth column) and the corresponding Figure (last column) are reported. (PDF) [file pone.0120984.s003.pdf]

experiment	measurement	genotype and condition	value (mean ± SD)	n	p-value	related Figure
era-1 mRNA distribution	mRNA levels				**A vs. P**	Figure 1I
		wild type A	11.5 ± 1.7	9	5.67×10^{-10}	
		wild type P	4.1 ± 0.9	9		
		era-1(RNAi) A	1.3 ± 0.2	12	0.326	
		era-1(RNAi) P	1.2 ± 0.2	12		
		par-3(RNAi) A	9.1 ± 2.9	10	0.907	
		par-3(RNAi) P	9 ± 2.9	10		
		mex-5(RNAi) A	9.5 ± 1.8	7	0.287	
		mex-5(RNAi) P	8.4 ± 1.8	7		
yfp mRNA distribution	mRNA levels				**A vs. P**	Figure 2G
		yfp-era-1[3'era-1] A	6.9 ± 0.7	9	1.03×10^{-7}	
		yfp-era-1[3'era-1] P	3.4 ± 0.9	9		
		yfp[3'era-1] A	2.7 ± 0.6	8	2×10^{-4}	
		yfp[3'era-1] P	1.9 ± 0.4	8		
		yfp-era-1[3'pie-1] A	3.2 ± 0.8	10	0.362	
		yfp-era-1[3'pie-1] P	2.9 ± 0.8	10		
YFP membrane enrichment	relative YFP levels				**control vs. RNAi-treated**	Figure 4E
		yfp-era-1[3'era-1] control A	2.1 ± 0.6	10	-	
		yfp-era-1[3'era-1] control P	0.8 ±0.3	10	-	
		yfp-era-1[3'era-1] mex-5/6(RNAi) A	0.5 ± 0.1	10	4.42×10^{-7}	
		yfp-era-1[3'era-1] mex-5/6(RNAi) P	0.5 ± 0.1	10	1.46×10^{-3}	
		yfp-era-1[3'era-1] par-3(RNAi) A	1.4 ± 0.5	9	9.32×10^{-3}	
		yfp-era-1[3'era-1] par-3(RNAi) P	1.3 ± 0.5	9	0.04	
		yfp-era-1[3'era-1] par-1(RNAi) A	2.2 ± 0.6	10	0.741	
		yfp-era-1[3'era-1] par-1(RNAi) P	2.1 ± 0.6	10	1.81×10^{-5}	
					**control vs. RNAi-treated**	Figure 4H
		yfp-era-1[3'pie-1] control A	3.9 ± 0.8	10	-	
		yfp-era-1[3'pie-1] control P	3.7 ± 0.9	10	-	
		yfp-era-1[3'pie-1] mex-5/6(RNAi) A	3.3 ± 1	8	0.113	
		yfp-era-1[3'pie-1] mex-5/6(RNAi) P	3.2 ± 0.8	8	0.205	
					**wild type vs. *era-1(tm6426)***	
era-1(tm6426) embryogenesis	embryonic lethality	wild type partial *par-3(RNAi)*	21.9 ± 18	8 exps.	-	Suppl. Fig. S1C
		era-1(tm6426) partial *par-3(RNAi)*	49.5 ± 16	8 exps.	4.6×10^{-3}	
		wild type partial *par-2(RNAi)*	17.9 ± 17.5	3 exps.	-	
		era-1(tm6426) partial *par-2(RNAi)*	55.7 ± 15.4	3 exps.	0.048	
					**control vs. *mex-5(RNAi)***	
qPCR	relative mRNA levels	*yfp-era-1[3'era-1]* control	7.3 ± 3.8	3 exps.	-	Suppl. Fig. S2B
		yfp-era-1[3'era-1] mex-5(RNAi)	4.7 ± 3.2	3 exps.	0.429	
		yfp[3'era-1] control	3.3 ± 1.3	3 exps.	-	
		yfp[3'era-1] mex-5(RNAi)	1.9 ±1.2	3 exps.	0.234	
		yfp-era-1[3'pie-1] control	3.7 ± 1.1	3 exps.	-	
		yfp-era-1[3'pie-1] mex-5(RNAi)	5.1 ± 1.7	3 exps.	0.284	
					**control vs. RNAi-treated**	
YFP membrane enrichment	relative YFP levels	*yfp-era-1[3'era-1]* control A	1.4 ± 0.4	11	-	Suppl. Fig. S2C
		yfp-era-1[3'era-1] control P	0.6 ± 0.2	11	-	
		yfp-era-1[3'era-1] mex-5(RNAi) A	0.9 ± 0.4	7	0.021	
		yfp-era-1[3'era-1] mex-5(RNAi) P	0.4 ± 0.1	7	0.012	
		yfp-era-1[3'era-1] mex-3(RNAi) A	1.4 ± 0.4	7	0.465	
		yfp-era-1[3'era-1] mex-3(RNAi) P	0.6 ± 0.1	7	0.124	

Supplemental Table S1
